# Aortic Root and Distal Arch Management During Type A Aortic Dissection Repair: Expanding Horizons

**DOI:** 10.21470/1678-9741-2021-0178

**Published:** 2022

**Authors:** Kaushalendra Rathore, Mark Newman

**Affiliations:** 1 Department of Cardiothoracic Surgery, Sir Charles Gairdner Hospital, Nedlands, Australia.

**Keywords:** Angina, Dissecting Aneurysm, Aorta, Cardiopulmonary Bypass, Perfusion, Decision

## Abstract

The management of Type A aortic dissection has evolved over a period of a decade or so, and contemporary reports are suggesting a paradigm shift from a conservative approach to complete excision of the diseased aorta including root and distal arch. Improved cardiopulmonary bypass perfusion techniques, better understanding of the cerebral perfusion, and wide-ranging obtainability of prosthetic conduits gave surgical teams numerous choices. With improving outcomes and maturing surgical techniques, surgeons are performing extensive resections of the diseased aorta, but there is no standard protocol as far as the extent of the proximal and distal diseased aortic tissue resection is concerned. Aortic root replacement is associated with good early- and long-term outcomes and proffered solution in young and stable patients, for that reason many busy centres are endorsing total arch replacement in complex distal aortic dissections. This systemic review is discussing contemporary literature and associated pros and cons during surgical decision-making for these high-risk cases.

**Table:** 

Abbreviations, Acronyms & Symbols			
ACP	= Antegrade cerebral perfusion	HAR	= Hemiarch replacement
AI	= Aortic insufficiency	IRAD	= International Registry of Aortic Dissection
AR	= Aortic regurgitation	MI	= Myocardial infarction
ARR	= Aortic root replacement	MOF	= Multi-organ failure
CABG	= Coronary artery bypass grafting	MPS	= Malperfusion syndrome
CPB	= Cardiopulmonary bypass	SCAR	= Supracoronary aortic replacement
CPR	= Cardiopulmonary resuscitation	SCI	= Spinal cord infarction
CT	= Computed tomography	SOV	= Sinus of Valsalva
CTD	= Connective tissue disorder	STJ	= Sinotubular junction
DHCA	= Deep hypothermic circulatory arrest	TAAD	= Type A aortic dissection
FET	= Frozen elephant trunk	TAR	= Total arch replacement
GERAADA	= German Registry for Acute Aortic Dissection	TEVAR	= Thoracic endovascular aortic repair

## INTRODUCTION

Various surgical units continue to aspire for a single-digit in-hospital mortality for type A aortic dissection (TAAD) surgery and are passionately trying to standardize the dissection repair techniques for predictable outcomes^[1]^. Leaving aside results from the high-volume aortic surgery hospitals, TAAD repair is still associated with significant mortality and morbidity (15-30%)^[2]^. Vastly variable outcomes in these patients can be because of extent of the dissection, malperfusion syndromes, aortic rupture, location of primary entry tear, haemodynamic instability, painless dissection and delayed clinical diagnosis, age, other comorbidities, and, finally, overall experience of the surgical team.

There continues the ongoing discussion of the pros and cons of conservative supracoronary aortic replacement (SCAR) *vs.* aortic root replacement (ARR) or hemiarch replacement (HAR) *vs.* total arch replacement (TAR) with or without antegrade stenting. Interestingly, opinions about operative management vary even amongst the experts^[3-6]^. These variations are not just limited to the surgical procedure only but to other steps, like inflow arterial cannulation site (femoral, axillary, central aortic, or innominate artery), temperature management (use of deep hypothermic circulatory arrest or without circulatory arrest), and cerebral perfusion strategies (no adjunctive, antegrade, or retrograde perfusion). For example, contemporary literature is showing that American surgeons use femoral artery as preferred inflow site, while European centres use femoral artery inflow only in 25% of the cases. Further, European surgeons use antegrade cerebral perfusion (ACP) in 69% of the time while the Society of Thoracic Surgeons data showed that ACP was used only in 44% of the cases in the Americas^[6]^.

The International Registry of Aortic Dissection (IRAD) described that the primary indication of emergency surgery is the “prevention of the death from TAAD rupture and is mainly accomplished by excision of the proximal initial entry tear with SCAR in majority of the cases” (59%)^[7]^. Mostly, ARR was performed using a valved conduit, while 6% of cases underwent valve-sparing procedure. Independent predictors of mortality in the IRAD analysis were aortic valve replacement, migrating chest pain, limb ischemia, hypotension, shock, and cardiac tamponade.

But, often by executing conservative surgery, surgeons might have a sense of not doing enough to address the issue of aortic events in the long-term follow-up^[5,8]^. We are presenting a concise review based on available literature with various arguments which could be involved in the thought processes during surgical decision-making.

## PROS AND CONS OF THE ESTABLISHED CLASSIFICATIONS

The incidence of aortic dissection in various published reports is between 4-5/100,000, and variability of the referral pattern is also a well-known phenomenon^[9]^. DeBakey et al.^[10]^ had proposed a classification of aortic dissection based on the anatomy of the entry tear and extent of the dissection, but this does not give prognostic guidance. On the other hand, the Stanford classification recognizes that prognosis is dependent on the involvement of the ascending aorta in the aortic dissection^[11]^. Drawback of both classifications is that neither the Stanford system nor the DeBakey system addresses dissections that originate from or extend proximally into the aortic arch but do not involve the ascending aorta. In the last decade, non-A non-B dissection cases have been recognised as a separate group where the entry tear is present in the aortic arch (11%) and, interestingly, 28% of these cases have a shared origin of the innominate and left carotid common artery (Bovine arch)^[12]^.

## NEWER CLASSIFICATIONS

DeBakey and Stanford classifications have many inherent lacunae, and to fill those information gaps various classifications have been proposed. The European Task Force included thoracic aortic pathology in their classification, and they have separated acute aortic syndrome in the five subclasses and advised it to be used beside the DeBakey or Stanford classification (classical dissection with intimal flap, intramural haematoma, discrete dissection, penetrating ulcer, and iatrogenic or traumatic dissection)^[13]^. We have discussed our review based on the Stanford classification as it is the most commonly used system in the clinical practise.

Booher et al.^[14]^ have described a classification from the IRAD data, which is based on the time period of the patient’s first presentation, and reported that overall survival was very well correlated to the time of presentation to the hospital. They described it as hyperacute (symptom onset to 24 hours), acute (2-7 days), subacute (8-30 days), and chronic (> 30 days). They found that overall survival was progressively lower through the four time periods.

Sievers et al.^[15,16]^ have proposed a classification for aortic dissection based on type, entry location and malperfusion status (or TEM), which can be used along with Stanford aortic dissection classification system. It reflects the extent of the disease process and helps in the prognostication of the early outcome. Type A is called when dissection is involving the ascending aorta with or without extension into the aortic arch and descending aorta. Type B is declared when dissection is involving the descending aorta, but not the aortic arch or ascending aorta. Type non-A non-B is a dissection involving the aortic arch and the descending aorta, but not the ascending aorta.

Sievers et al.^[16]^ have reported location of the entry tear in the ascending aorta among 80% of the patients, while 7% of the patients had tear in the aortic arch (type A E2), and no entry was identified in 9% of the patients (type A E0). The in-hospital mortality rate was 16%, 5%, and 8% in patients with type A, B, and non-A non-B, respectively.

With continually growing endovascular interventions, many investigators are in the opinion that these age-old recognized classifications might be good enough for the surgical decision-making, but it is suboptimum for the planning of radiological interventions. The “DISSECT” classification (acronym for Duration of disease, Intimal tear location, Size of the dissected aorta, Segmental Extent of the involvement, and Thrombus within the false lumen) is used by various vascular interventionists during the decision-making for complex endovascular stenting^[17]^.

### Take-home Message

With increasing overall international experience, more and more classifications and criteria are coming into the clinical practise, and most of them can be used in adjunct with the Stanford or DeBakey classification.

## THE IMPORTANCE OF RISK STRATIFICATION FOR TAAD CASES

Routine risk stratification can be a daunting task as > 90% of patients presenting with TAAD usually fail to meet the guidelines for elective ascending replacement before dissection onset, and nearly two thirds of the non-marfanoid patients with spontaneous TAAD have a non-dilated ascending aorta before dissection onset^[18]^.

Recent publications from IRAD data have revealed similar in-hospital mortality in ARR compared to the SCAR surgery, but the postoperative myocardial infarction was more frequent in patients who underwent ARR (4.4% *vs.* 1.9%), and it was associated with high in-hospital mortality rates (57.9% *vs.* 16.3%)^[18]^. These findings suggest that risk stratification becomes an important step in determining the extent of the aortic resection and, eventually, prognosticating the outcomes. Ghoreishi and Wise et al.^[19]^ have reported a simple algorithm based on the serum lactate, creatinine, and liver function tests to calculate a “mortality score”. TAAD patients with high mortality score (> 20) but without evidence of severe cardiogenic shock would benefit from aortic fenestration before central aortic repair. Their group endorses that this algorithm predicts operative mortality with high accuracy, compared to the Penn classification.

The Penn classification is based on the malperfusion assessment, and patients were classified as: class A (absence of ischemia with hemodynamic stability), class B (localized visceral ischemia, including stroke, renal failure, paraplegia, extremity ischemia, and mesenteric ischemia), class C (generalized ischemia — here associated with coronary malperfusion and heart failure), or class B and C (both localized and general ischemia). Various recent publications have suggested that Penn classes B, C, and combined B+C are independent risk factors for a poor outcome^[20]^.

Similarly, preoperative cardiopulmonary resuscitation (CPR) is recognized as a significant risk for early postoperative mortality^[5]^. Uehara et al.^[21]^ have reported that CPR of > 15 minutes is a contraindication for the dissection surgery and cautioned to reconsider going ahead if spontaneous return of the circulation does not happen after pericardiotomy. They had reasonable results in patients under 10 minutes of CPR, but beyond that all patients had neurological deficits after the index aortic surgery.

Sun et al.^[22]^ and their group in China, in another busy centre, have proposed a classification for triaging TAAD patients based on the severity of the aortic root lesions (A1, A2, and A3) and extent of the dissection into the ascending aorta, aortic arch, and distal thoracic aorta (C: complex; S: simple). The patient was classified from A1, when sinotubular junction, aortic sinus, and aortic valve were intact, to A3, when the patient had severe aortic regurgitation (AR) with total destruction of the aortic root, with root measuring > 50 mm. Similarly, the patient was classified as complex (C) when the primary tear was located in the transverse arch or the descending aorta, there was aneurysm formation in the aortic arch or the distal aorta (40 mm), involvement of the arch, aneurysm formation, occlusion of the brachiocephalic artery, Marfan syndrome, and sleeve-like striping (intussusception).

### Take-home Message

Routine risk stratification can be a daunting task as > 90% of patients presenting with TAAD usually fail to meet the guidelines for elective ascending replacement before dissection onset, and nearly two thirds of the non-marfanoid patients with spontaneous TAAD have a non-dilated ascending aorta before dissection onset^[23]^.

## AORTIC DISSECTION TEAM AND SURGICAL EXPERIENCE

High-volume centres can consolidate outcomes by delegating surgeons as point of contact for these complex surgeries, but it cannot be generalized universally^[6]^. Andersen et al.^[24]^ have reported significant reduction in the in-hospital mortality rate after making a TAAD focused team (33.7% before and 7.7% after a six-year interval), and it also helped in growing overall referrals to the institute.

### Take-home Message

Although these improvements in the contemporary results could be a composite outcome of many subtle elements, like infrastructure improvements, better understanding of surgical techniques, improved intensive care management, and rehabilitation, the importance of technical expertise of the surgical team cannot be overstated^[3,4]^.

## MALPERFUSION: COMPLICATED OR UNCOMPLICATED

Preoperative malperfusion and acidosis have been known predictors of high mortality in TAAD patients, and severe metabolic acidosis (base deficit ≥ -10) was uniformly fatal in patients with systemic malperfusion (brain only, coronary only, visceral only, and visceral plus extremity)^[25]^.

The incidence of cerebral ischemia, gut ischemia, and limb ischemia rates are 10-14%, 15%, and 10%, respectively, in the data from IRAD^[7]^. All these patients are at very high risk of morbidity and mortality after the index surgery.

Czerny et al.^[26]^ have analysed the German Registry for Acute Aortic Dissection (or GERAADA) database and suggested that malperfusion of multiple organs carries poor outcomes. They found a linear correlation between the number of malperfused organs and mortality (10% increase in the mortality per any additional organ involved, 31% if patients had at least two organs involved, while 44% if patients had three organs involved). Based on these findings, their group have divided TAAD cases into “uncomplicated” (no clinical or imaging signs of malperfusion) and “complicated” (malperfusion present).

Postoperative persistent malperfusion was more common in patients with preoperative cerebral malperfusion, comatose state, visceral and peripheral malperfusion, dissection extending into the neck vessels, and coronary circulation. Among all these, visceral malperfusion is the worst subgroup, with a very high mortality, and opening discussion whether distal stenting should be done prior to the cardiac surgery^[26]^.

Yang et al.^[27]^ have suggested that patients of TAAD with malperfusion syndrome have a risk of dying from organ failure higher than the risk of dying from aortic rupture, and surgeons have to balance the risk of operative mortality in these complicated cases (30% if index surgery was performed first) or the 4% risk of aortic rupture (if fenestration was performed first)^[28]^. But these recommendations cannot be generalised as delay in the dissection surgery in patients with malperfusion is a known risk factor of in-hospital mortality (5-23%).

Involvement of the supra-aortic branches in TAAD cases varies from 28% to 73%, and it significantly increases the probabilities of postoperative stroke. Incidence of postoperative stroke ranges between 8-17% after TAAD repair, and malperfusion to the right carotid artery and preoperative CPR are known independent predictors of the stroke^[29]^. Presence of coma or ischemic stroke preoperatively should not represent a contraindication to the index surgery. However, documentation of the hemorrhagic stroke on brain computed tomography (CT) scan, a prolonged time from initial presentation to the surgery, or the presence of significant concomitant comorbidities, such as advanced age, further increase the operative risk and should be considered when determining whether or not to offer an operation^[30]^.

### Take-home Message

Patients with severe proximal malperfusion syndrome (acute AR, tamponade, impending rupture, and coronary ischemic changes) and distal arch or limb vessel-related malperfusion (stroke, coma, intimal intussusception, and limb ischemia) should be treated with the urgent index aortic surgery and then can be referred later to the vascular surgeons for management of visceral malperfusion^[31]^.Abdominal malperfusion is a totally separate subset of patients and should be managed by a multidisciplinary “aortic team”.

## AORTIC ROOT ISSUES: ROUTINE AORTIC ROOT REPLACEMENT OR TRIAGE DECISION-MAKING

Many authors are impersonating distal aortic resection protocols and endorsing ARR in all cases except where root is pristine^[8]^. The arguments in favour of replacing aortic root are valve-sparing procedures and availability of better prosthetic conduits, which may reduce incidence of aortic root related reoperations. Sun et al.^[22]^ based their surgical decision-making on a classification, and it simplified surgical choices, bringing down 30-day mortality to the single digits. Ikena et al.^[32]^ have presented a retrospective analysis of 339 cases of SCAR for TAAD and found that 40% of patients have commissural detachment at the time of index surgery. Their analysis strongly suggested that a dilated sinus of Valsalva (SOV) and commissural detachment were significant risk factors for aortic root-related reoperations and deaths during the follow-up. They have found a linear relationship between the number of commissures detached and severity of AR in the follow-up.

Nishida et al.^[33]^ have reported mortality rates of 12.5% and 4.7% in cases undergoing AAR *vs.* no ARR, respectively, in their retrospective analysis and indicated that preoperative shock was as an independent predictor of operative death amongst both groups. Although the five-year survival was not significantly different among both groups (85% *vs.* 92%), the late aortic root events (redo surgery, severe AR, pseudoaneurysm, dilatation of SOV) were significantly higher in the non-ARR group (11.6%). Their multivariate analysis showed that dissection of two or more SOVs is associated with late aortic root events, and they supported the use of ARR in these cases. The presence of an intimal tear in the root, extensive dissection of the root, dissection of coronary arteries, dilatation of SOV > 45 mm, young age, Marfan syndrome, bicuspid aortic valve, aortic insufficiency, and previous aortic valve replacement are factors in favor of ARR. Likewise, Coselli et al.^[34]^ have presented an insightful overview of the contemporary practice in TAAD repair and endorsed that safety should be the first objective and that SCAR with HAR are sufficient in most of the patients.

Connective tissue disorder patients and especially patients with Marfan syndrome present at least 20 years early with vascular complications compared to the non-Marfan cohort^[35]^. Freedom from need for aortic root reoperation in patients who underwent primarily a composite valved graft or valve-sparing ARR procedure was 95±3%, 88±5%, and 79±5% and in patients who underwent supracoronary ascending replacement it was 83±9%, 60±13%, and 20±16% at five, 10, and 20 years, respectively. ARR or aortic root repair is highly recommended in Marfan syndrome patients because supracoronary ascending replacement is associated with a high need (> 40%) for root reintervention.

### Take-home Message

The incidence of late proximal reoperation after SCAR is between 3-9% in various studies, and major reasons of reoperation were SOV dilatation, pseudoaneurysm formation, and recurrent dissections. The mean growth rate of the aortic root after SCAR was 0.6 mm/year, and it becomes significant in patients with dilated baseline SOV^[33]^. The freedom from reoperation steadily decreases as time passes, and at the five-year mark, 65-72% of the patients may require some intervention, so, leaving behind a significant diseased segment might be detrimental for the long-term follow-up^[22,32,33,36]^.

## DISTAL AORTIC ISSUES

### Total Arch Replacement *vs.* Hemiarch Replacement

For the patients with tear in the ascending aorta and normal-caliber aortic arch without distal malperfusion, the standard surgical repair involves a HAR with an open distal anastomosis under deep hypothermic arrest (Table 1). Elimination of the proximal aortic tissue up to the orifice of the innominate artery and an aggressive bevel of the lesser curvature of the arch extending distally to the level of the ligamentum arteriosum are the hallmarks of a true HAR.

**Table 1:** Management strategies.

No	Site of TAAD	Indications	Simple decision-making	Complex decision-making	Possible consequences
1	STJ	Intimal tear in STJ	SCAR	Add hemiarch with DHCA	Stroke, bleeding
2	Aortic root: No AI With severe AI	Root > 4.5 cm CTD Unrepairable tear at root	Prosthetic aortic root replacement	Yacoub procedure David procedure	Prolonged CPB time, ischemic time, and reinterventions Postoperative MI
3	Coronary artery malperfusion	Dissection into arteries	Prosthetic root replacement	CABG or Cabrol procedure	Extensive surgery, MI
4	Distal ascending aorta (zone 0)	Entry tear Aorta > 4 cm	Hemiarch	Total arch and debranching	Prolonged CPB time, ischemic time, reintervention, paraplegia, and combined vascular interventions
5	Arch and distal aorta (zone 1 to 3)	MPS Aorta > 4 cm Entry tear	Total arch (zone 3) or partial debranching (zones 1 and 2)	Complete debranching, classical elephant trunk graft or antegrade stenting using frozen elephant trunk stent graft	Multiple teams involved, delay in decision-making, cost, and expertise
6	Brain malperfusion	Stroke, coma	Index aortic surgery first. Followed by vascular interventions	Vascular or coronary interventions first	Unsure about cognitive functions, debilitating stroke
7	Visceral malperfusion	Gut or limb ischemia	Vascular intervention first	Hybrid management	Shock, metabolic acidosis and low cardiac output, aortic rupture

AI=aortic insufficiency; CABG=coronary artery bypass grafting; CPB=cardiopulmonary bypass; CTD=connective tissue disorder; DHCA=deep hypothermic circulatory arrest; MI=myocardial infarction; MPS=malperfusion syndrome; SCAR=supracoronary aortic replacement; STJ=sinotubular junction; TAAD=type A aortic dissection

The possible benefits of extended resection are excluding primary intimal tears beyond the ascending aorta, excision of the re-entry tears in the distal aorta, facilitating re-expansion of the distal true lumen, and promoting false lumen obliteration. In approximately 70% of patients with TAAD, dissection flap extends beyond the ascending aorta and involves the aortic arch, and at least one entry tear was detected in 80% patients on the pre-discharge CT scan^[37]^.

Unfavorable remodeling after supracoronary ascending aorta replacement with aortic valve resuspension is well known in the mid- to long-term follow-up, and De Paulis et al.^[38]^ have reported that 36% of patients presented with aortic root dilatation of > 10%, and in 56% of the patients, AR progressed from mild to severe at a five-year follow-up. There was no significant difference in the postoperative stroke or perioperative mortality between HAR and TAR (zone 1/2/3) group when arch branch vessels were dissected without malperfusion^[39]^.

Based on the review of 38 studies and 2,140 patients, Smith et al.^[40]^ have proposed a classification for the “extended arch repair” and they have organized it around (1) the extent of surgical aortic resection (total arch *vs.* hemiarch) and (2) method and timing of descending thoracic aortic stent deployment (during circulatory arrest *vs.* normothermic perfusion with use of fluoroscopy). They have divided these patients in four groups: TAR standard elephant trunk without descending thoracic aortic stent grafting, TAR and descending thoracic aortic stent grafting with frozen stent graft placed under circulatory arrest, HAR and descending thoracic aortic stent grafting with the stent graft placed under circulatory arrest, and TAR with stent graft placed after coming off cardiopulmonary bypass and with the use of fluoroscopy to identify landing zones. They have reported mortality between 8.5-11% with very good mid-term outcomes.

Eusanio et al.^[41]^ have recommended that younger patients with intimal tear in the arch or distal aorta should be considered for TAR. Although the long-term follow-up has failed to prove any significant benefits of TAR compared to the conservative HAR, freedom from reoperation at seven-years follow-up were 71% *vs.* 85%, respectively. The motive behind this aggressive approach is to resect all diseased aortic tissue to limit the distal false lumen patency and aid in favorable distal aortic remodeling.

A systemic review has described no significant difference in the in-hospital mortality (3.6-24% *vs.* 3.85-29%) and neurological events between HAR *vs.* TAR cases^[2]^. TAR patients had significantly higher incidence of renal failure, longer operation time, reopening for bleeding, and ventilation time. Although the rate of distal aortic reinterventions was higher in the HAR group compared to the TAR group (7.3% *vs.* 3.3%), it was not statistically significant.

Norton et al.^[42]^ have recently reported a series of 276 cases where HAR was performed in TAAD patients and suggested that HAR is adequate in the patients without cerebral malperfusion syndrome (with or without dissection of aortic arch branches). There was no survival difference among both groups (aortic arch branches dissected *vs.* not involved) at an eight-year follow-up, but the intervention rate was high in the patients with neck vessel involvement (19% *vs.* 4%).

Yang et al.^[43]^ have presented a retrospective series comparing HAR with aggressive arch replacement (zones 2 and 3 resections) and found that there were no significant differences in perioperative outcomes, 30-day mortality (5.3% *vs.* 7.3%, respectively), and operative mortality among both groups. Good results in their series were mainly because of a dedicated “aortic team” and aggressive vascular intervention of the patients with systemic malperfusion, or in the cases with arch > 4 cm, entry tear in the arch or distal intimal flap could not be completely resected with TAR approach.

Omura et al.^[44]^ have reported early and late outcomes following HAR or TAR surgery and showed that preoperative CPR and visceral malperfusion were two significant risk factors for the in-hospital mortality. Various groups have reported 12-24% mortality in the first five years after the index surgery, and the reasons proposed are distal aortic dilatation and rupture or malperfusion requiring further interventions. These are the main reasons for endorsing “aortic tear excision” policy and performing TAR.

Eusanio et al.^[45]^ reviewed TAR using either E-vita Open plus (JOTEC, Gmbh, Hechihngen, Germany) or Thoroflex (Vascutek, Scotland) and reported acceptable in-hospital mortality (10%) and morbidity (stroke, 4.8%; spinal cord injury, 4.3%). Although their review does not reflect on very long-term survival data, it does report high rates of thrombosis of the distal false lumen (89%) and again stresses on the strict selection criteria before considering any aggressive surgical option. Apart from complex primary and re-entry intimal tears involving distal arch and thoracic aorta, other factors required to be considered are patient’s age, comorbidities, presence of distal arch aneurysm, distal thoracic aorta diameter > 35 mm, false lumen diameter > 20 mm, proximal intimal tear diameter > 10 mm, and presence of connective tissue disorder. During TAR with antegrade stent deployment, the risk of paraplegia and spinal cord infarction (SCI) are catastrophic complications along with a longer cardiopulmonary bypass time, aortic cross-clamping time, and cerebral perfusion time, and all these factors should be considered in the surgical decision-making. A pooled analysis showed overall mortality of 8.8% in cases of TAR with frozen elephant trunk (FET) deployment, where 53.2% of the cases were done in emergency situations^[46]^. Reported SCI was 4.7% of the cases, and it was higher when stent graft length was > 15 cm and beyond T8 vertebrae.

In a contemporary review, Pacini et al.^[47]^ have reported good outcomes of antegrade stenting of the descending aorta during TAAD repair. They have also quoted partial or complete thrombosis of the false lumen after first year of the index surgery, but this thrombosis was not uniform and frequently occlusion in false lumen was seen in the supra diaphragmatic thoracic aorta only. However, they have concluded that FET procedure is justified only in selected patients with less comorbidity and in patients with severe distal aortic arch malperfusion syndrome.

Sun et al.^[48]^ have reported a technique to treat complex TAAD and aneurysms by deploying a distal novel stented graft (MicroPort Medical Co Ltd, Shanghai, China) and performing TAR using a tetrafurcated graft with implantation.

### Take-home Message

TAR without distal aorta intervention might leave intimal tear behind in 20-30% of the patients and does not address distal malperfusion issues. TAD with the deployment of FET might be the quickest way to pay attention about the distal aortic issues, but it is done without fluoroscopy and landing zone may be missed. HAR can be a good surgical option in Sun’s A1 and A2 subclassification or with surgeons with less surgical experience.

## ENDOVASCULAR STENTING FOR TAAD

TAAD can present with a wide spectrum of clinical and radiological variations, and, occasionally, patients are too high risk for any surgical intervention. These are the subset of cases where thoracic endovascular aortic repair (TEVAR) can be offered in specialized centres, as the reported anatomic suitability for TEVAR in TAAD is between 32%-50% of patients on the CT scan^[49]^. The goals of TEVAR are similar to the surgical goal, that is, to cover the origin of the intimal tear in order to prevent aortic rupture as well as to reduce pressure and promote thrombosis of the false lumen^[50,51]^. TEVAR is contraindicated if there are severe aortic valve regurgitation, aortic root dissection, or in the presence of connective tissue disorder. Anatomical requirements for ascending aortic TEVAR are proximal and distal landing zones length > 10 mm and intimal tear > 5 mm proximal to the innominate artery. The distal landing zone can be extended by performing a left carotid to right carotid artery bypass if coverage of the innominate artery is required to achieve adequate distal seal.

Ye et al.^[52]^ have reported good success rate of TEVAR technique (97%) with a 30-day mortality rate of 6.7% in cases of TAAD at a follow-up of 36 months. Noticeably, 71% of the patients had positive remodeling and developed complete thrombosis of the false lumen.

Contemporary literature endorses sufficient resection of the distal diseased aortic segment and debranching of arch vessels with the deployment of the FET to obliterate distal false lumen (covering zones 0, 1, and 2 in the aorta) and keep true lumen open with the stent support^[53]^.

Chronic dissections (non-operated dissection survivors or postoperative residual disease) are another complex and tedious subset of patients and require proper planning for reasonable outcomes. The thickened and stiff dissection flap makes it less compliable and compresses true lumen. The tortuousness of the aorta may create a kink at the junction between the arch and the descending aorta. During conventional first-stage elephant trunk procedures, this situation can pose a problem if the elephant trunk graft is compressed within the true lumen, thereby creating a pseudocoarctation. These patients can be treated using various FET grafts and available modifications with good outcomes, but post-surgery neurological complications are well known events, especially if graft is kept 15-20 cm long^[54]^.

### Take-home Message

FET helps by immediately expanding the true lumen and excludes communications between the lumina in the proximal aorta, stopping malperfusion to the brain and visceral circulation^[55]^. Secondly, it facilitates favorable remodeling and reduces the incidence of future reinterventions. These interventions have their own complications (*e.g.*, retrograde TAAD, aortic rupture, trauma to the aortic valve and to coronary arteries, stroke, distal embolization, endoleaks) and should be performed in centres with enough experience. An important message from this review is that inclusion of entry tear in the excision is the main factor to stop the progression of the distal disease and to improve favorable aortic remodeling (Table 2).

**Table 2:** Summary of contemporary literature in TAAD patients.

Authors	Number of patients	Type of study	Postoperative complications	Risk factors for poor outcomes	30-day mortality	Conclusions
Poon et al.^[2]^, 2016	2,221 patients from 14 studies	Systemic review	In-hospital mortality in hemiarch and TAR groups	TAR group had longer cross-clamping bypass time	Big variation in mortality rate in different studies	High volume centres have good TAR results; if entry is in root and ascending aorta, then hemiarch is adequate
Uehara et al.^[21]^, 2021	34 cases required CPR before surgery (out of a total of 519)	Retrospective single-centre	Aortic rupture was the most common cause of CPR (61.8%), followed by coronary malperfusion (13.5%)	Preoperative neurological deficit, duration of CPR		CPR duration > 15 minutes may be a contraindication for surgery
Czerny et al.^[26]^, 2015	2,137 cases 717 had malperfusion)	GERAADA analysis	Cerebral malperfusion (6.8%), visceral malperfusion (3.8%)	Peripheral malperfusion, coronary malperfusion, preoperative coma, tear in descending aorta, age	Overall (16.9%), one-organ malperfusion (21.3%)	Type of dissection and number of organs affected in malperfusion decide the outcome
Yang et al.^[27]^, 2018	597 cases (137 treated with stent first and then index surgery approach)	Retrospective single-centre	Aortic rupture (4%)	Multi-organ malperfusion, index surgery first approach	First decade of follow-up (21%), second decade (10.7%)	Risk of dying from MOF was 6.6 times higher compared to the aortic rupture; stable patient with malperfusion can be managed with stent first approach
Dumfarth et al.^[29]^, 2018	303 cases underwent TAAD repair	Retrospective single-centre	Overall stroke rate (15.8%), stroke in preoperative CPR cases (18.8%), no CPR cases (3.5%)	Preoperative CPR, bovine arch, and malperfusion increase the incidence of stroke	Overall (13.2%), patients with stroke (22.8%)	Preoperative CPR and preoperative malperfusion syndromes are independent predictors of postoperative stroke
Ikeno et al.^[32]^, 2021	339 cases underwent SCAR	Retrospective single-centre	At 5-year follow-up, aortic root-related redo surgery (2.5%), overall deaths (14.5%)	Dilated SOV, number of commissural detachments	13.6%	SOV and commissural detachment are predictors of unfavorable outcomes
Nishida et al.^[33]^, 2016	316 cases underwent ARR during TAAD surgery	Retrospective single-centre	Aortic root event (11.6%) in the non-ARR group	Dissection of > 2 SOV	ARR group (12.5%), non-ARR group (4.7%)	ARR reduces future aortic root events
Conzelmann et al.^[37]^, 2015	2,137 TAAD cases treated with surgery	Multi-centre, GERAADA data	Mortality of TAAD surgery for septuagenarians (16%) and for octogenarians (35%)	Age, preoperative coma, CPR, multi-organ malperfusion	16.9%	Mortality risk in TAAD patients depends on clinical presentation
Nortan et al.^[39]^, 2020	399 cases underwent TAAD surgery, 190 had arch vessel dissection	Retrospective single-centre	Overall, there were no significant differences in major postoperative outcomes between hemiarch and zone 1/2/3 arch groups; 5-year survival: hemiarch (79%) *vs.* zone 1/2/3 cases (85%)	Acute MI and cardiogenic shock, hemiarch group had higher reoperation rate at 5-year follow-up (23%)	Hemiarch group (7%), zone 1/2/3 group (5%)	Branch alone involvement without malperfusion should not be an indication of debranching
Eusanio et al.^[41]^, 2014	240 cases (53 total arch replacements, 187 hemiarch)	Retrospective single-centre	5-year survival for arch and hemiarch group was 65% and 60%, respectively	Distal entry tear, cardiogenic shock	Arch group (22%), hemiarch group (24%)	Aortic and patients’ characteristics greatly influenced the extent of the aortic replacement; 20% of the patients underwent arch replacement
Yang et al.^[43]^, 2019	Hemiarch (322 cases), TAR (150 cases)	Retrospective single-centre	Stroke rate was the same in both groups (7% each), 10-year survival was similar (hemiarch 70% *vs.* TAR 72%)	Arch > 4 cm, intimal tear in the arch, and branch malperfusion were indications for debranching	Mortality was similar in both groups (hemiarch 5.3% *vs.* TAR 7.3%)	Both hemiarch and TAR are appropriate in the selective cases
Omura et al.^[44]^, 2016	109 hemiarch cases and 88 TAR cases	Retrospective single-centre	5-year event rates were low in TAR group	30% of TAR group cases had entry in the arch	Hemiarch (14.7%), TAR (10.2%)	Acceptable TAR mortality with good long-term survival; preoperative CPR and visceral malperfusion are bad indicators
Preventza et al.^[46]^, 2020	3,154 following FET	Meta-analysis	SCI (4.7%), stroke (7.6%)	Higher SCI rate in stent length > 15 cm or coverage of T8 vertebrae	8.8%	Unclear outcome, stent length < 10 cm was associated with less SCI
Ma et al.^[48]^	Sun’s procedure	Retrospective single-centre	Stroke (19.8%)	SCI (2.5%)	7.8%	Higher mortality seen in patients with stroke, SCI, and low cardiac output

ARR=aortic root replacement; CPR=cardiopulmonary resuscitation; FET=frozen elephant trunk; GERAADA=German Registry for Acute Aortic Dissection; MI=myocardial infarction; MOF=multi-organ failure; SCAR=supracoronary aortic replacement; SCI=spinal cord infarction; SOV=sinus of Valsalva; TAAD=type A aortic dissection; TAR=total arch replacement

## CONCLUSION

Proper HAR is sufficient in most of the critically sick patients with TAAD as there is no difference in the long-term survival among HAR *vs.* TAR patients. Although surgeons can salvage most patients with supracoronary ascending aorta replacement and resuspension of aortic valve, ARR is indicated in selected cases, especially in young patients with severe root and aortic valve pathology. Complex TAAD involving arch and distal aorta should be treated with adequate aortic resection and antegrade stent deployment by the surgeons with adequate experience. Cerebral, coronary, or limb malperfusion cases should be operated immediately with index aortic surgery, while patients with severe visceral ischemia should be managed with a team multidisciplinary approach.
